# *N*,*N*-Diethyl-2-(4-methyl­benzene­sulfonamido)­benzamide

**DOI:** 10.1107/S160053681204264X

**Published:** 2012-10-20

**Authors:** Maria Altamura, Valentina Fedi, Rossano Nannicini, Paola Paoli, Patrizia Rossi

**Affiliations:** a Chemistry Department, Menarini Ricerche S.p.A., Via dei Sette Santi 3, I-50131 Firenze, Italy; b Dip. Energetica "Sergio Stecco", University of Firenze, Via S. Marta 3, I-50139 Firenze, Italy

## Abstract

The asymmetric unit of the title compound, C_18_H_22_N_2_O_3_S, contains two mol­ecules, exhibiting similar conformations [C—S—N—C torsion angles of −82.2 (2) and −70.4 (2)°, and dihedral angles between the mean planes of the aromatic rings of 56.6 (6) and 51.6 (6)° in mol­ecules I and II, respectively]. However, the two independent mol­ecules show distinctly different hydrogen-bonding patterns. In the crystal, molecules I form inversion dimers *via* pairs of N—H⋯O hydrogen bonds, whereas for molecules II the N—H⋯O hydrogen bond is intramolecular. The hydrogen-bonded dimers of I further propagate along the *b-*axis direction through π–π inter­actions [the distance between ring centroids is 3.8424 (8) Å].

## Related literature

For the synthesis of the title compound, see: Bakker *et al.* (1997 ▶); Kaul *et al.* (2002 ▶). For the biological activity of compounds having the sulfonamide –SO_2_NH– group, see: Lu & Tucker (2007 ▶); Tappe *et al.* (2008 ▶); Chegwidden *et al.* (2000 ▶); Purushottamachar *et al.* (2008 ▶). For structural and conformational studies of mol­ecules featuring the sulfonamide moiety, see: Parkin *et al.* (2008 ▶); Perlovich *et al.* (2009 ▶, 2011 ▶); Altamura *et al.* (2009 ▶); Vega-Hissi *et al.* (2011 ▶).
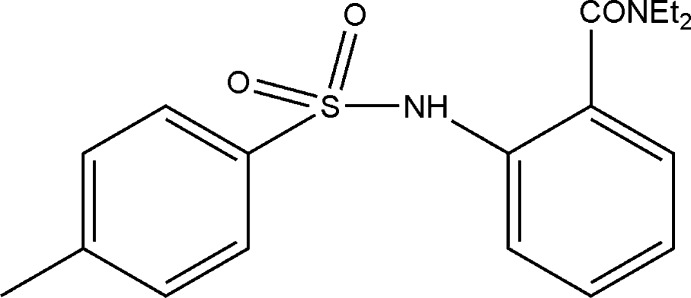


## Experimental

### 

#### Crystal data

C_18_H_22_N_2_O_3_S
*M* *_r_* = 346.43
Triclinic, 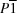

*a* = 9.4674 (6) Å
*b* = 12.2882 (9) Å
*c* = 16.0569 (12) Å
α = 108.426 (7)°
β = 97.357 (6)°
γ = 100.245 (6)°
*V* = 1709.7 (2) Å^3^
*Z* = 4
Mo *K*α radiation
μ = 0.21 mm^−1^
*T* = 150 K
0.54 × 0.43 × 0.38 mm

#### Data collection

Oxford Diffraction Xcalibur3 CCD diffractometer
Absorption correction: multi-scan (*ABSPACK* in *CrysAlis RED*; Oxford Diffraction, 2006 ▶) *T*_min_ = 0.894, *T*_max_ = 1.000
17890 measured reflections
7512 independent reflections
4728 reflections with *I* > 2σ(*I*)
*R*_int_ = 0.025

#### Refinement

*R*[*F* ^2^ > 2σ(*F* ^2^)] = 0.045
*wR*(*F* ^2^) = 0.118
*S* = 0.96
7512 reflections
441 parameters
H atoms treated by a mixture of independent and constrained refinement
Δρ_max_ = 0.42 e Å^−3^
Δρ_min_ = −0.32 e Å^−3^

### 

Data collection: *CrysAlis CCD* (Oxford Diffraction, 2006 ▶); cell refinement: *CrysAlis CCD*; data reduction: *CrysAlis RED* (Oxford Diffraction, 2006 ▶); program(s) used to solve structure: *SIR97* (Altomare *et al.*, 1999 ▶); program(s) used to refine structure: *SHELXL97* (Sheldrick, 2008 ▶); molecular graphics: *ORTEP-3* (Farrugia, 1997 ▶); software used to prepare material for publication: *SHELXL97*, *WinGX* (Farrugia, 1999 ▶) and *PARST* (Nardelli, 1995 ▶).

## Supplementary Material

Crystal structure: contains datablock(s) I, global. DOI: 10.1107/S160053681204264X/ld2075sup1.cif

Structure factors: contains datablock(s) I. DOI: 10.1107/S160053681204264X/ld2075Isup2.hkl

Supplementary material file. DOI: 10.1107/S160053681204264X/ld2075Isup3.cml

Additional supplementary materials:  crystallographic information; 3D view; checkCIF report

## Figures and Tables

**Table 1:** Hydrogen-bond geometry (Å, °)

*D*—H⋯*A*	*D*—H	H⋯*A*	*D*⋯*A*	*D*—H⋯*A*
N1′—H*N*1′⋯O3′	0.81 (2)	2.15 (2)	2.809 (2)	139 (2)
N1—H*N*1⋯O3^i^	0.86 (2)	2.15 (2)	2.969 (2)	159 (2)

Symmetry code: (i) 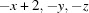.

**Fig. 1.**
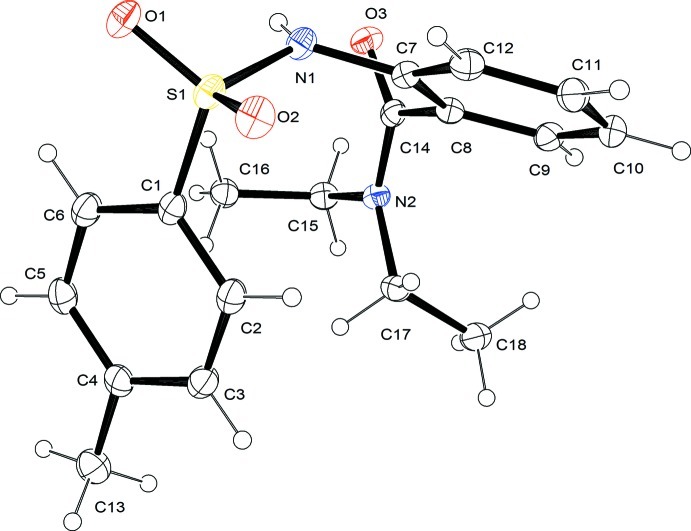
The symmetrically independent molecule I (molecule II has a similar shape and the same labelling scheme). Displacement ellipsoids are drawn at the 30% probability level.

**Fig. 2.**
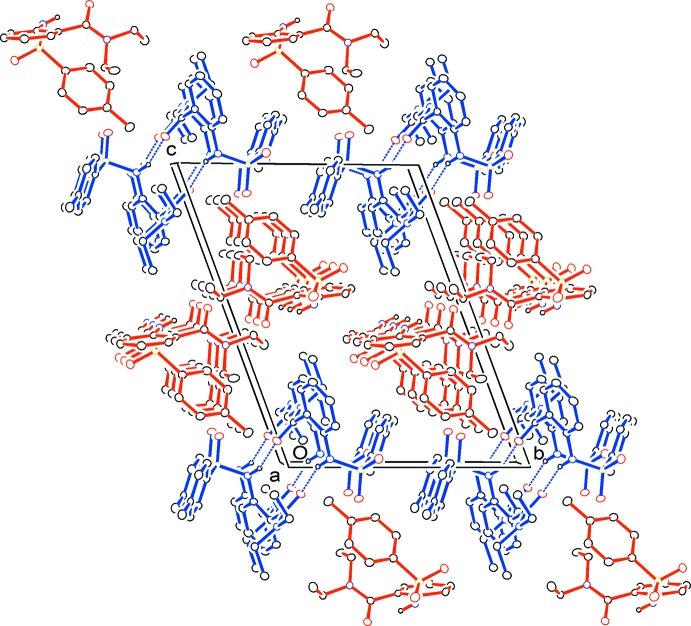
Crystal structure of the title compound as viewed along the a-axis (molecules I are shown in blue and molecules II - in red). Intermolecular NH···O hydrogen bonding is shown as dashed lines.

## supplementary crystallographic information

### Comment

The sulfonamide moiety is a common pharmacophore in many biologically active compounds, such as HIV inhibitors (Lu & Tucker, 2007), antimicrobial drugs (Tappe *et al.*, 2008), carbonic anhydrase inhibitors (Chegwidden *et al.*, 2000), and anti-tumor agents (Purushottamachar *et al.*, 2008). Because the structural and conformational properties of a compound usually are related to its biological properties, their study would provide useful information to design new effective drugs. In this regard, there are many recent publications reporting structural data on related sulfonamides (Parkin *et al.*, 2008, Altamura *et al.*, 2009, Perlovich *et al.*, 2009, Perlovich *et al.*, 2011,Vega-Hissi *et al.*, 2011).

The asymmetric unit of the title compound contains two independent molecules, I and II, which are almost superimposable (Table 1). As expected, a staggered conformation about the N—S bond is adopted, with the N lone pair bisecting the OŜO angle, and with the p orbital at the *ipso* carbon bisecting the same angle (Table 1, Fig. 1). The sulfonamide nitrogen atom is almost planar-trigonal in molecule I (Σ<N=355 (1)°), while in II it is definitely more pyramidal (Σ<N=341 (1)°). The conformation of molecule II is stabilized by an intramolecular H-bond involving the H atom of the sulfonamide grouping (HN1') and the oxygen atom O3' of the amide moiety (Table 2). In the crystal packing, molecules I form dimers instead, which are held together by a couple of N—H···O=C hydrogen bonds (Table 2, Fig. 2). Dimers propagate along the *b* axis direction through π-π stacking interactions involving two symmetry related C1—C6 rings (centroid-centroid distance 3.8424 (8) Å, symmetry code: -*x* + 2, -*y* + 1, -*z*). No further significant intermolecular interactions are present in the crystal structure.

### Experimental

For the synthesis of the title compound, see: Bakker *et al.* (1997); Kaul *et al.*, (2002). Crystals of *N*,*N*-diethyl-2-(4-methylphenylsulfonamido)benzamide suitable for single-crystal X-ray diffraction analysis were obtained by slow evaporation of an ethanol/water solution of *N*,*N*-diethyl-2-(4-methylphenylsulfonamido)benzamide.

### Refinement

The N—H H atoms were located in the Fourier difference map and their coordinates were refined with *U*_iso_(H) = 1.2*U*_eq_(N). All other H atoms were positioned using idealized geometry, and refined using a riding model with *U*_iso_(H) 1.2 times *U*_eq_(C) (1.5 for methyl H atoms).

### Crystal data

**Table:**

C_18_H_22_N_2_O_3_S	*Z* = 4
*M**_r_* = 346.43	*F*(000) = 736
Triclinic, *P*1	*D*_x_ = 1.346 Mg m^−^^3^
*a* = 9.4674 (6) Å	Mo *K*α radiation, λ = 0.71073 Å
*b* = 12.2882 (9) Å	θ = 4.1–28.6°
*c* = 16.0569 (12) Å	µ = 0.21 mm^−^^1^
α = 108.426 (7)°	*T* = 150 K
β = 97.357 (6)°	Parallelepiped, colourless
γ = 100.245 (6)°	0.54 × 0.43 × 0.38 mm
*V* = 1709.7 (2) Å^3^	

### Data collection

**Table:**

Oxford Diffraction Xcalibur3 CCD diffractometer	7512 independent reflections
Radiation source: Enhance (Mo) X-ray Source	4728 reflections with *I* > 2σ(*I*)
Graphite monochromator	*R*_int_ = 0.025
Detector resolution: 16.4547 pixels mm^-1^	θ_max_ = 28.7°, θ_min_ = 4.1°
ω scans	*h* = −12→11
Absorption correction: multi-scan (*ABSPACK* in *CrysAlis RED*; Oxford Diffraction, 2006)	*k* = −15→16
*T*_min_ = 0.894, *T*_max_ = 1.000	*l* = −21→21
17890 measured reflections	

### Refinement

**Table:**

Refinement on *F*^2^	Primary atom site location: structure-invariant direct methods
Least-squares matrix: full	Secondary atom site location: difference Fourier map
*R*[*F*^2^ > 2σ(*F*^2^)] = 0.045	Hydrogen site location: inferred from neighbouring sites
*wR*(*F*^2^) = 0.118	H atoms treated by a mixture of independent and constrained refinement
*S* = 0.96	*w* = 1/[σ^2^(*F*_o_^2^) + (0.0677*P*)^2^] where *P* = (*F*_o_^2^ + 2*F*_c_^2^)/3
7512 reflections	(Δ/σ)_max_ = 0.001
441 parameters	Δρ_max_ = 0.42 e Å^−^^3^
0 restraints	Δρ_min_ = −0.32 e Å^−^^3^

### Special details

**Table:**

Geometry. All s.u.'s (except the s.u. in the dihedral angle between two l.s. planes) are estimated using the full covariance matrix. The cell s.u.'s are taken into account individually in the estimation of s.u.'s in distances, angles and torsion angles; correlations between s.u.'s in cell parameters are only used when they are defined by crystal symmetry. An approximate (isotropic) treatment of cell s.u.'s is used for estimating s.u.'s involving l.s. planes.
Refinement. Refinement of *F*^2^ against ALL reflections. The weighted *R*-factor *wR* and goodness of fit *S* are based on *F*^2^, conventional *R*-factors *R* are based on *F*, with *F* set to zero for negative *F*^2^. The threshold expression of *F*^2^ > 2σ(*F*^2^) is used only for calculating *R*-factors(gt) *etc*. and is not relevant to the choice of reflections for refinement. *R*-factors based on *F*^2^ are statistically about twice as large as those based on *F*, and *R*- factors based on ALL data will be even larger.

### Fractional atomic coordinates and isotropic or equivalent isotropic displacement parameters (Å^2^)

**Table:**

	*x*	*y*	*z*	*U*_iso_*/*U*_eq_	
S1	1.22768 (5)	0.28575 (4)	−0.00356 (3)	0.02970 (14)	
O1	1.18234 (15)	0.23441 (12)	−0.09887 (8)	0.0365 (3)	
O2	1.36507 (14)	0.36978 (12)	0.03457 (9)	0.0365 (3)	
O3	1.03929 (14)	−0.01020 (12)	0.08734 (9)	0.0329 (3)	
N1	1.23284 (17)	0.17480 (15)	0.03138 (10)	0.0278 (4)	
HN1	1.158 (2)	0.1167 (18)	0.0058 (13)	0.033*	
N2	0.98318 (16)	0.13823 (13)	0.19374 (10)	0.0257 (4)	
C1	1.0900 (2)	0.35179 (16)	0.03962 (12)	0.0268 (4)	
C2	1.1191 (2)	0.42734 (17)	0.12797 (13)	0.0311 (5)	
H2	1.2108	0.4429	0.1638	0.037*	
C3	1.0114 (2)	0.47931 (17)	0.16265 (13)	0.0331 (5)	
H3	1.0315	0.5296	0.2220	0.040*	
C4	0.8731 (2)	0.45770 (17)	0.11021 (13)	0.0308 (5)	
C5	0.8479 (2)	0.38376 (18)	0.02171 (14)	0.0348 (5)	
H5	0.7570	0.3698	−0.0146	0.042*	
C6	0.9537 (2)	0.33011 (18)	−0.01428 (13)	0.0334 (5)	
H6	0.9339	0.2802	−0.0737	0.040*	
C7	1.30951 (19)	0.18780 (16)	0.11830 (12)	0.0262 (4)	
C8	1.23673 (19)	0.14737 (16)	0.17723 (12)	0.0257 (4)	
C9	1.3163 (2)	0.15919 (17)	0.26008 (12)	0.0304 (5)	
H9	1.2691	0.1312	0.2991	0.037*	
C10	1.4645 (2)	0.21191 (18)	0.28524 (13)	0.0343 (5)	
H10	1.5162	0.2206	0.3411	0.041*	
C11	1.5345 (2)	0.25127 (18)	0.22645 (13)	0.0332 (5)	
H11	1.6340	0.2868	0.2431	0.040*	
C12	1.4591 (2)	0.23881 (17)	0.14310 (13)	0.0305 (5)	
H12	1.5082	0.2644	0.1037	0.037*	
C13	0.7557 (2)	0.51226 (19)	0.14887 (15)	0.0397 (5)	
H13A	0.6650	0.4798	0.1062	0.060*	
H13B	0.7826	0.5960	0.1626	0.060*	
H13C	0.7444	0.4959	0.2026	0.060*	
C14	1.0785 (2)	0.08570 (17)	0.14925 (12)	0.0250 (4)	
C15	0.82675 (19)	0.07980 (17)	0.16405 (13)	0.0293 (4)	
H15A	0.7766	0.1054	0.2133	0.035*	
H15B	0.8154	−0.0048	0.1472	0.035*	
C16	0.7572 (2)	0.10750 (18)	0.08525 (13)	0.0326 (5)	
H16A	0.6554	0.0680	0.0677	0.049*	
H16B	0.8054	0.0810	0.0360	0.049*	
H16C	0.7666	0.1911	0.1020	0.049*	
C17	1.0220 (2)	0.25858 (16)	0.25954 (12)	0.0291 (4)	
H17A	0.9527	0.3020	0.2443	0.035*	
H17B	1.1183	0.2979	0.2561	0.035*	
C18	1.0228 (2)	0.26194 (18)	0.35513 (13)	0.0373 (5)	
H18A	1.0486	0.3424	0.3951	0.056*	
H18B	1.0930	0.2208	0.3712	0.056*	
H18C	0.9272	0.2247	0.3594	0.056*	
S1'	0.36428 (5)	0.62169 (4)	0.38496 (3)	0.02947 (14)	
O1'	0.25700 (14)	0.66660 (13)	0.43239 (9)	0.0389 (4)	
O2'	0.34408 (14)	0.49807 (12)	0.34048 (9)	0.0356 (3)	
O3'	0.71780 (14)	0.88703 (11)	0.52660 (9)	0.0346 (3)	
N1'	0.51694 (17)	0.66700 (15)	0.46038 (11)	0.0279 (4)	
HN1'	0.534 (2)	0.7376 (18)	0.4848 (14)	0.033*	
N2'	0.81608 (16)	0.90225 (13)	0.40820 (10)	0.0263 (4)	
C1'	0.39078 (18)	0.69322 (17)	0.30717 (12)	0.0273 (4)	
C2'	0.45199 (19)	0.64421 (18)	0.23389 (13)	0.0297 (4)	
H2'	0.4787	0.5727	0.2251	0.036*	
C3'	0.4727 (2)	0.70303 (18)	0.17430 (13)	0.0316 (5)	
H3'	0.5144	0.6706	0.1256	0.038*	
C4'	0.43286 (19)	0.80900 (18)	0.18556 (13)	0.0314 (5)	
C5'	0.3715 (2)	0.85577 (19)	0.25892 (14)	0.0369 (5)	
H5'	0.3431	0.9265	0.2671	0.044*	
C6'	0.3517 (2)	0.79973 (18)	0.32016 (13)	0.0345 (5)	
H6'	0.3124	0.8333	0.3697	0.041*	
C7'	0.64714 (19)	0.63062 (17)	0.43794 (12)	0.0246 (4)	
C8'	0.76801 (19)	0.71269 (16)	0.43306 (12)	0.0251 (4)	
C9'	0.8955 (2)	0.67471 (17)	0.41748 (12)	0.0279 (4)	
H9'	0.9765	0.7277	0.4145	0.034*	
C10'	0.9044 (2)	0.56046 (18)	0.40639 (13)	0.0319 (5)	
H10'	0.9906	0.5369	0.3963	0.038*	
C11'	0.7839 (2)	0.48063 (17)	0.41038 (13)	0.0316 (5)	
H11'	0.7894	0.4033	0.4025	0.038*	
C12'	0.6555 (2)	0.51581 (17)	0.42602 (13)	0.0293 (4)	
H12'	0.5750	0.4620	0.4285	0.035*	
C13'	0.4544 (2)	0.8733 (2)	0.12051 (14)	0.0437 (6)	
H13D	0.5286	0.8482	0.0885	0.066*	
H13E	0.4842	0.9567	0.1528	0.066*	
H13F	0.3642	0.8559	0.0789	0.066*	
C14'	0.76384 (19)	0.84015 (17)	0.45779 (12)	0.0263 (4)	
C15'	0.8238 (2)	1.02962 (17)	0.44075 (13)	0.0318 (5)	
H15C	0.9033	1.0690	0.4203	0.038*	
H15D	0.8465	1.0589	0.5057	0.038*	
C16'	0.6845 (2)	1.0613 (2)	0.40996 (15)	0.0457 (6)	
H16D	0.6968	1.1454	0.4334	0.069*	
H16E	0.6056	1.0244	0.4312	0.069*	
H16F	0.6624	1.0344	0.3457	0.069*	
C17'	0.8356 (2)	0.85147 (18)	0.31525 (12)	0.0305 (5)	
H17C	0.7752	0.8800	0.2772	0.037*	
H17D	0.8015	0.7664	0.2952	0.037*	
C18'	0.9930 (2)	0.8811 (2)	0.30377 (14)	0.0443 (6)	
H18D	0.9980	0.8455	0.2420	0.066*	
H18E	1.0534	0.8515	0.3400	0.066*	
H18F	1.0270	0.9651	0.3220	0.066*	

### Atomic displacement parameters (Å^2^)

**Table:**

	*U*^11^	*U*^22^	*U*^33^	*U*^12^	*U*^13^	*U*^23^
S1	0.0316 (3)	0.0309 (3)	0.0249 (3)	0.0009 (2)	0.0054 (2)	0.0110 (2)
O1	0.0445 (8)	0.0397 (9)	0.0227 (7)	0.0037 (7)	0.0059 (6)	0.0109 (7)
O2	0.0314 (7)	0.0366 (8)	0.0390 (8)	−0.0025 (6)	0.0063 (6)	0.0153 (7)
O3	0.0342 (7)	0.0305 (8)	0.0278 (7)	0.0052 (6)	0.0064 (6)	0.0029 (7)
N1	0.0266 (8)	0.0268 (10)	0.0249 (9)	−0.0003 (7)	0.0016 (7)	0.0071 (8)
N2	0.0283 (8)	0.0233 (9)	0.0245 (8)	0.0048 (7)	0.0064 (7)	0.0069 (7)
C1	0.0323 (10)	0.0229 (10)	0.0241 (10)	−0.0005 (8)	0.0027 (8)	0.0113 (9)
C2	0.0320 (11)	0.0308 (12)	0.0274 (11)	0.0020 (9)	−0.0003 (9)	0.0111 (9)
C3	0.0401 (12)	0.0313 (12)	0.0259 (11)	0.0053 (9)	0.0046 (9)	0.0097 (10)
C4	0.0377 (11)	0.0225 (11)	0.0340 (12)	0.0039 (9)	0.0046 (9)	0.0150 (10)
C5	0.0301 (11)	0.0346 (12)	0.0383 (12)	0.0048 (9)	−0.0028 (9)	0.0159 (10)
C6	0.0372 (11)	0.0312 (12)	0.0268 (11)	0.0021 (9)	−0.0005 (9)	0.0090 (10)
C7	0.0280 (10)	0.0261 (11)	0.0225 (10)	0.0079 (8)	0.0044 (8)	0.0052 (9)
C8	0.0284 (10)	0.0219 (10)	0.0263 (10)	0.0073 (8)	0.0053 (8)	0.0069 (9)
C9	0.0367 (11)	0.0311 (12)	0.0254 (10)	0.0115 (9)	0.0082 (9)	0.0096 (9)
C10	0.0358 (11)	0.0357 (12)	0.0276 (11)	0.0132 (9)	−0.0023 (9)	0.0063 (10)
C11	0.0238 (10)	0.0357 (12)	0.0338 (11)	0.0071 (9)	0.0004 (9)	0.0055 (10)
C12	0.0294 (10)	0.0304 (11)	0.0317 (11)	0.0075 (9)	0.0089 (9)	0.0093 (10)
C13	0.0403 (12)	0.0358 (13)	0.0450 (13)	0.0087 (10)	0.0081 (10)	0.0168 (11)
C14	0.0305 (10)	0.0246 (11)	0.0216 (10)	0.0052 (8)	0.0050 (8)	0.0108 (9)
C15	0.0265 (10)	0.0307 (11)	0.0315 (11)	0.0053 (8)	0.0098 (9)	0.0108 (9)
C16	0.0302 (10)	0.0376 (12)	0.0325 (11)	0.0104 (9)	0.0082 (9)	0.0137 (10)
C17	0.0333 (11)	0.0253 (11)	0.0313 (11)	0.0103 (9)	0.0095 (9)	0.0101 (9)
C18	0.0493 (13)	0.0328 (12)	0.0299 (11)	0.0142 (10)	0.0081 (10)	0.0086 (10)
S1'	0.0235 (2)	0.0323 (3)	0.0314 (3)	0.0011 (2)	0.0062 (2)	0.0119 (2)
O1'	0.0278 (7)	0.0482 (9)	0.0444 (9)	0.0060 (7)	0.0164 (7)	0.0190 (8)
O2'	0.0335 (7)	0.0288 (8)	0.0369 (8)	−0.0040 (6)	0.0007 (6)	0.0093 (7)
O3'	0.0431 (8)	0.0284 (8)	0.0290 (8)	0.0027 (6)	0.0121 (7)	0.0067 (7)
N1'	0.0286 (9)	0.0247 (9)	0.0289 (9)	0.0024 (8)	0.0072 (7)	0.0090 (8)
N2'	0.0269 (8)	0.0237 (9)	0.0255 (9)	−0.0003 (7)	0.0012 (7)	0.0096 (7)
C1'	0.0190 (9)	0.0322 (11)	0.0278 (10)	0.0023 (8)	0.0008 (8)	0.0100 (9)
C2'	0.0238 (10)	0.0305 (11)	0.0316 (11)	0.0048 (8)	0.0037 (9)	0.0082 (10)
C3'	0.0273 (10)	0.0388 (13)	0.0276 (11)	0.0041 (9)	0.0049 (9)	0.0119 (10)
C4'	0.0240 (10)	0.0355 (12)	0.0316 (11)	−0.0006 (9)	−0.0020 (9)	0.0144 (10)
C5'	0.0406 (12)	0.0336 (12)	0.0381 (12)	0.0125 (10)	0.0042 (10)	0.0138 (11)
C6'	0.0345 (11)	0.0387 (13)	0.0312 (11)	0.0134 (10)	0.0085 (9)	0.0100 (10)
C7'	0.0242 (9)	0.0288 (11)	0.0199 (9)	0.0034 (8)	0.0026 (8)	0.0095 (9)
C8'	0.0266 (10)	0.0249 (11)	0.0200 (10)	−0.0006 (8)	0.0006 (8)	0.0079 (9)
C9'	0.0262 (10)	0.0291 (11)	0.0264 (10)	0.0008 (8)	0.0035 (8)	0.0105 (9)
C10'	0.0272 (10)	0.0349 (12)	0.0312 (11)	0.0062 (9)	0.0034 (9)	0.0097 (10)
C11'	0.0366 (11)	0.0260 (11)	0.0304 (11)	0.0069 (9)	0.0023 (9)	0.0090 (9)
C12'	0.0298 (10)	0.0268 (11)	0.0299 (11)	−0.0005 (8)	0.0024 (9)	0.0133 (9)
C13'	0.0447 (13)	0.0475 (14)	0.0386 (13)	0.0027 (11)	0.0016 (11)	0.0216 (11)
C14'	0.0241 (10)	0.0251 (11)	0.0256 (10)	0.0005 (8)	0.0008 (8)	0.0076 (9)
C15'	0.0361 (11)	0.0240 (11)	0.0303 (11)	−0.0016 (9)	0.0020 (9)	0.0092 (9)
C16'	0.0511 (14)	0.0364 (13)	0.0467 (14)	0.0125 (11)	0.0002 (11)	0.0128 (11)
C17'	0.0304 (10)	0.0321 (12)	0.0246 (10)	−0.0013 (9)	0.0012 (9)	0.0102 (9)
C18'	0.0385 (12)	0.0611 (16)	0.0281 (11)	0.0002 (11)	0.0080 (10)	0.0141 (11)

### Geometric parameters (Å, º)

**Table:**

S1—O1	1.4315 (13)	S1'—O2'	1.4248 (14)
S1—O2	1.4324 (13)	S1'—O1'	1.4311 (14)
S1—N1	1.6358 (17)	S1'—N1'	1.6446 (17)
S1—C1	1.758 (2)	S1'—C1'	1.758 (2)
O3—C14	1.235 (2)	O3'—C14'	1.246 (2)
N1—C7	1.435 (2)	N1'—C7'	1.434 (2)
N1—HN1	0.86 (2)	N1'—HN1'	0.81 (2)
N2—C14	1.350 (2)	N2'—C14'	1.346 (2)
N2—C17	1.469 (2)	N2'—C15'	1.470 (2)
N2—C15	1.470 (2)	N2'—C17'	1.475 (2)
C1—C2	1.388 (3)	C1'—C6'	1.383 (3)
C1—C6	1.390 (3)	C1'—C2'	1.391 (3)
C2—C3	1.381 (3)	C2'—C3'	1.382 (3)
C2—H2	0.9300	C2'—H2'	0.9300
C3—C4	1.395 (3)	C3'—C4'	1.384 (3)
C3—H3	0.9300	C3'—H3'	0.9300
C4—C5	1.386 (3)	C4'—C5'	1.386 (3)
C4—C13	1.500 (3)	C4'—C13'	1.509 (3)
C5—C6	1.384 (3)	C5'—C6'	1.380 (3)
C5—H5	0.9300	C5'—H5'	0.9300
C6—H6	0.9300	C6'—H6'	0.9300
C7—C12	1.393 (3)	C7'—C12'	1.381 (3)
C7—C8	1.400 (3)	C7'—C8'	1.413 (2)
C8—C9	1.393 (3)	C8'—C9'	1.393 (3)
C8—C14	1.493 (2)	C8'—C14'	1.498 (3)
C9—C10	1.385 (3)	C9'—C10'	1.377 (3)
C9—H9	0.9300	C9'—H9'	0.9300
C10—C11	1.378 (3)	C10'—C11'	1.388 (3)
C10—H10	0.9300	C10'—H10'	0.9300
C11—C12	1.382 (3)	C11'—C12'	1.388 (3)
C11—H11	0.9300	C11'—H11'	0.9300
C12—H12	0.9300	C12'—H12'	0.9300
C13—H13A	0.9600	C13'—H13D	0.9600
C13—H13B	0.9600	C13'—H13E	0.9600
C13—H13C	0.9600	C13'—H13F	0.9600
C15—C16	1.513 (3)	C15'—C16'	1.508 (3)
C15—H15A	0.9700	C15'—H15C	0.9700
C15—H15B	0.9700	C15'—H15D	0.9700
C16—H16A	0.9600	C16'—H16D	0.9600
C16—H16B	0.9600	C16'—H16E	0.9600
C16—H16C	0.9600	C16'—H16F	0.9600
C17—C18	1.521 (3)	C17'—C18'	1.518 (3)
C17—H17A	0.9700	C17'—H17C	0.9700
C17—H17B	0.9700	C17'—H17D	0.9700
C18—H18A	0.9600	C18'—H18D	0.9600
C18—H18B	0.9600	C18'—H18E	0.9600
C18—H18C	0.9600	C18'—H18F	0.9600
			
O1—S1—O2	119.57 (8)	O2'—S1'—O1'	120.27 (8)
O1—S1—N1	105.49 (8)	O2'—S1'—N1'	107.60 (8)
O2—S1—N1	107.66 (8)	O1'—S1'—N1'	104.98 (8)
O1—S1—C1	108.16 (9)	O2'—S1'—C1'	108.68 (9)
O2—S1—C1	107.99 (9)	O1'—S1'—C1'	107.76 (9)
N1—S1—C1	107.40 (8)	N1'—S1'—C1'	106.80 (8)
C7—N1—S1	123.27 (13)	C7'—N1'—S1'	120.56 (13)
C7—N1—HN1	118.4 (13)	C7'—N1'—HN1'	110.6 (14)
S1—N1—HN1	113.0 (13)	S1'—N1'—HN1'	110.0 (15)
C14—N2—C17	124.10 (15)	C14'—N2'—C15'	117.36 (15)
C14—N2—C15	117.84 (15)	C14'—N2'—C17'	125.41 (16)
C17—N2—C15	117.24 (14)	C15'—N2'—C17'	115.80 (15)
C2—C1—C6	120.03 (18)	C6'—C1'—C2'	120.19 (18)
C2—C1—S1	119.44 (14)	C6'—C1'—S1'	119.29 (14)
C6—C1—S1	120.52 (15)	C2'—C1'—S1'	120.51 (15)
C3—C2—C1	119.89 (18)	C3'—C2'—C1'	119.20 (19)
C3—C2—H2	120.1	C3'—C2'—H2'	120.4
C1—C2—H2	120.1	C1'—C2'—H2'	120.4
C2—C3—C4	121.13 (18)	C2'—C3'—C4'	121.49 (18)
C2—C3—H3	119.4	C2'—C3'—H3'	119.3
C4—C3—H3	119.4	C4'—C3'—H3'	119.3
C5—C4—C3	117.83 (18)	C3'—C4'—C5'	118.20 (18)
C5—C4—C13	121.32 (18)	C3'—C4'—C13'	121.76 (18)
C3—C4—C13	120.85 (18)	C5'—C4'—C13'	120.04 (19)
C6—C5—C4	122.02 (18)	C6'—C5'—C4'	121.47 (19)
C6—C5—H5	119.0	C6'—C5'—H5'	119.3
C4—C5—H5	119.0	C4'—C5'—H5'	119.3
C5—C6—C1	119.07 (18)	C5'—C6'—C1'	119.44 (18)
C5—C6—H6	120.5	C5'—C6'—H6'	120.3
C1—C6—H6	120.5	C1'—C6'—H6'	120.3
C12—C7—C8	119.84 (17)	C12'—C7'—C8'	120.49 (17)
C12—C7—N1	119.24 (17)	C12'—C7'—N1'	118.96 (16)
C8—C7—N1	120.90 (16)	C8'—C7'—N1'	120.45 (16)
C9—C8—C7	118.94 (17)	C9'—C8'—C7'	118.05 (17)
C9—C8—C14	120.59 (17)	C9'—C8'—C14'	121.72 (16)
C7—C8—C14	120.36 (16)	C7'—C8'—C14'	119.60 (16)
C10—C9—C8	121.10 (18)	C10'—C9'—C8'	121.49 (17)
C10—C9—H9	119.4	C10'—C9'—H9'	119.3
C8—C9—H9	119.4	C8'—C9'—H9'	119.3
C11—C10—C9	119.21 (19)	C9'—C10'—C11'	119.66 (18)
C11—C10—H10	120.4	C9'—C10'—H10'	120.2
C9—C10—H10	120.4	C11'—C10'—H10'	120.2
C10—C11—C12	121.06 (18)	C12'—C11'—C10'	120.24 (18)
C10—C11—H11	119.5	C12'—C11'—H11'	119.9
C12—C11—H11	119.5	C10'—C11'—H11'	119.9
C11—C12—C7	119.83 (18)	C7'—C12'—C11'	120.06 (17)
C11—C12—H12	120.1	C7'—C12'—H12'	120.0
C7—C12—H12	120.1	C11'—C12'—H12'	120.0
C4—C13—H13A	109.5	C4'—C13'—H13D	109.5
C4—C13—H13B	109.5	C4'—C13'—H13E	109.5
H13A—C13—H13B	109.5	H13D—C13'—H13E	109.5
C4—C13—H13C	109.5	C4'—C13'—H13F	109.5
H13A—C13—H13C	109.5	H13D—C13'—H13F	109.5
H13B—C13—H13C	109.5	H13E—C13'—H13F	109.5
O3—C14—N2	122.48 (16)	O3'—C14'—N2'	121.77 (17)
O3—C14—C8	119.72 (16)	O3'—C14'—C8'	118.54 (16)
N2—C14—C8	117.80 (16)	N2'—C14'—C8'	119.61 (16)
N2—C15—C16	111.74 (15)	N2'—C15'—C16'	113.69 (16)
N2—C15—H15A	109.3	N2'—C15'—H15C	108.8
C16—C15—H15A	109.3	C16'—C15'—H15C	108.8
N2—C15—H15B	109.3	N2'—C15'—H15D	108.8
C16—C15—H15B	109.3	C16'—C15'—H15D	108.8
H15A—C15—H15B	107.9	H15C—C15'—H15D	107.7
C15—C16—H16A	109.5	C15'—C16'—H16D	109.5
C15—C16—H16B	109.5	C15'—C16'—H16E	109.5
H16A—C16—H16B	109.5	H16D—C16'—H16E	109.5
C15—C16—H16C	109.5	C15'—C16'—H16F	109.5
H16A—C16—H16C	109.5	H16D—C16'—H16F	109.5
H16B—C16—H16C	109.5	H16E—C16'—H16F	109.5
N2—C17—C18	113.09 (16)	N2'—C17'—C18'	113.74 (15)
N2—C17—H17A	109.0	N2'—C17'—H17C	108.8
C18—C17—H17A	109.0	C18'—C17'—H17C	108.8
N2—C17—H17B	109.0	N2'—C17'—H17D	108.8
C18—C17—H17B	109.0	C18'—C17'—H17D	108.8
H17A—C17—H17B	107.8	H17C—C17'—H17D	107.7
C17—C18—H18A	109.5	C17'—C18'—H18D	109.5
C17—C18—H18B	109.5	C17'—C18'—H18E	109.5
H18A—C18—H18B	109.5	H18D—C18'—H18E	109.5
C17—C18—H18C	109.5	C17'—C18'—H18F	109.5
H18A—C18—H18C	109.5	H18D—C18'—H18F	109.5
H18B—C18—H18C	109.5	H18E—C18'—H18F	109.5
			
C1—S1—N1—C7	−82.2 (2)	C1'—S1'—N1'—C7'	−70.4 (2)
HN1—N1—S1—O1	−44 (1)	HN1'—N1'—S1'—O1'	−54 (2)
C7—N1—S1—O2	33.9 (2)	C7'—N1'—S1'—O2'	46.1 (2)
C6—C1—S1—O1	11.7 (2)	C6'—C1'—S1'—O1'	20.8 (2)

### Hydrogen-bond geometry (Å, º)

**Table:**

*D*—H···*A*	*D*—H	H···*A*	*D*···*A*	*D*—H···*A*
N1′—H*N*1′···O3′	0.81 (2)	2.15 (2)	2.809 (2)	139 (2)
N1—H*N*1···O3^i^	0.86 (2)	2.15 (2)	2.969 (2)	159 (2)

Symmetry code: (i) −*x*+2, −*y*, −*z*.
